# Preoperative standing–supine difference in joint line convergence angle predicts postoperative alignment change after closing‐wedge high tibial osteotomy

**DOI:** 10.1002/jeo2.70904

**Published:** 2026-09-07

**Authors:** Mitsuhiko Kubo, Kazuhiro Uenaka, Hitomi Fujikawa, Tsutomu Maeda, Kosuke Kumagai, Yuki Nosaka, Sadafumi Horikawa, Shinji Imai

**Affiliations:** ^1^ Department of Sports and Musculoskeletal Pain Medicine Shiga University of Medical Science Otsu Japan; ^2^ Department of Orthopaedic Surgery Shiga University of Medical Science Otsu Japan

**Keywords:** alignment, closing‐wedge, high tibial osteotomy, joint line convergence angle, preoperative planning

## Abstract

**Purpose:**

The aim of this study was to identify preoperative determinants of postoperative change in joint line convergence angle (JLCA) after closing‐wedge high tibial osteotomy (CWHTO), comparing standing‐ and supine‐based radiographic references for preoperative planning.

**Methods:**

Consecutive patients with medial compartmental knee disorders who underwent CWHTO between February 2009 and September 2017 were retrospectively reviewed. Patients with anterior cruciate ligament reconstruction, unavailable radiographs or non‐measurable radiographs were excluded. JLCA was measured on standing, supine and valgus/varus stress radiographs. Postoperative change in JLCA (cJLCA) was calculated from preoperative supine to postoperative standing JLCA (cJLCA [pre‐supine to post‐stand]) and from preoperative standing to postoperative standing JLCA (cJLCA [pre‐stand to post‐stand]). The preoperative standing‐supine difference in JLCA (dJLCA) was also calculated. Associations between cJLCA and demographic and radiographic variables were evaluated using univariate and multivariable analyses.

**Results:**

After excluding patients with anterior cruciate ligament reconstruction (*n* = 1), unavailable radiographs (*n* = 7) or non‐measurable radiographs (*n* = 4), 40 patients were included. The cohort included 29 women and 11 men, with a mean age of 60.3 years (range, 39–77 years); postoperative radiographs were obtained at a mean of 9.9 months (range, 2–14 months). Univariate analysis showed that dJLCA was positively correlated with cJLCA (pre‐supine to post‐stand) (*ρ* = 0.52, *p* = 0.001) and negatively correlated with cJLCA (pre‐stand to post‐stand) (*ρ* = −0.43, *p* = 0.007). In multivariable analysis, dJLCA remained independently associated with both outcomes (*B* = 0.51, *p* = 0.006; *B* = −0.49, *p* = 0.008, respectively).

**Conclusions:**

The preoperative standing‐supine difference in JLCA was the primary determinant of postoperative change in JLCA after CWHTO. A greater standing‐supine difference was associated with greater undercorrection when supine radiographs were used as the planning reference, but with greater overcorrection when standing radiographs were used as the planning reference.

**Level of Evidence:**

Level IV.

## Graphical abstract

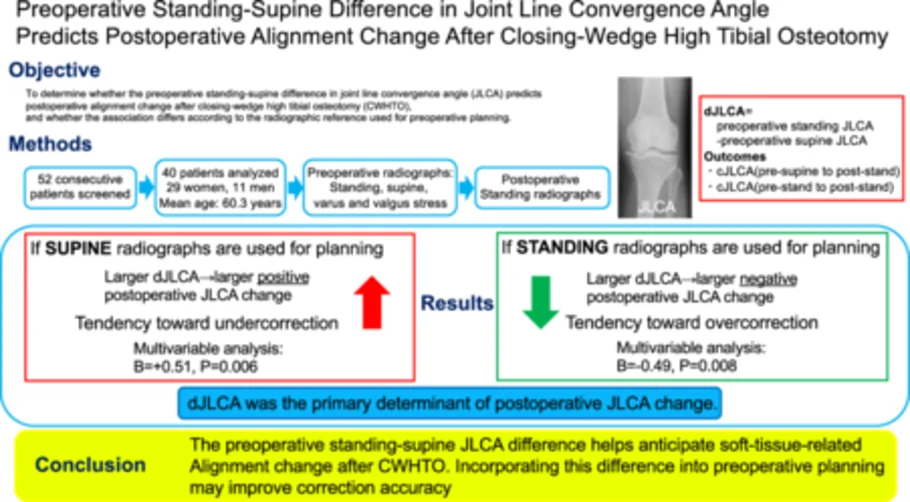

**Abbreviations**
BMI: body mass index
cJLCA: change in the joint line convergence angle
CWHTO: closing‐wedge high tibial osteotomy
dJLCA: difference in the joint line convergence angle between the preoperative standing and supine positions
HKA: hip–knee–ankle
HTO: high tibial osteotomy
JLCA: joint line convergence angle
LDFA: lateral distal femoral angle
MPTA: medial proximal tibial angle
OWHTO: open‐wedge high tibial osteotomy

## INTRODUCTION

High tibial osteotomy (HTO) is an established procedure for the treatment of knee disorders associated with coronal malalignment. Based on deformity analysis, HTO is performed to correct pathological lower‐limb alignment and optimize load distribution across the knee joint. In patients with medial compartmental knee disorders and varus alignment, valgus‐producing HTO shifts excessive load from the medial to the lateral compartment [5, 9]. Long‐term outcomes depend on achieving the intended postoperative alignment [6, 15]. Therefore, accurate preoperative planning is essential.

Despite careful technique, postoperative alignment does not always match the preoperative plan [11, 13, 14, 19, 20, 21, 22, 23, 25]. This discrepancy is driven not only by the achieved bony correction but also by postoperative changes in soft‐tissue balance. Postoperative change in joint line convergence angle (cJLCA) can contribute to unintended alignment and correction error [11, 13, 19, 23, 25]. However, conventional planning primarily targets the intended change in medial proximal tibial angle (MPTA) and typically does not account for postoperative cJLCA [11, 13, 19, 20, 23, 25]. Identifying preoperative determinants of cJLCA is therefore important to anticipate soft‐tissue–related changes and improve planning accuracy [10].

JLCA varies with limb position and loading conditions [7, 26]. Although postoperative evaluation is usually based on standing whole‐leg radiographs, the optimal choice of standing versus supine radiographs for preoperative planning remains controversial. Because cJLCA is referenced to the preoperative radiographic condition, both the magnitude and determinants of cJLCA may differ between standing‐ and supine‐based planning [7, 21, 26].

Accordingly, the aim of this study was to identify preoperative determinants of cJLCA after closing‐wedge HTO, using both standing‐ and supine‐based planning. The primary aim was to determine which preoperative radiographic factors, including the standing–supine difference in JLCA (dJLCA) and varus/valgus stress JLCA, were independently associated with cJLCA. The secondary aim was to compare these associations between standing‐ and supine‐based planning. It was hypothesized that dJLCA would be the principal determinant of cJLCA and that its association with cJLCA would differ depending on the radiographic condition used for preoperative planning.

## MATERIALS AND METHODS

### Ethics approval

This retrospective study was approved by the Ethics Committee of Shiga University of Medical Science (No. R2025‐007) and was conducted in accordance with the Declaration of Helsinki. The requirement for informed consent was waived by the Institutional Review Board because of the retrospective observational study design using existing clinical and radiographic data, and an opt‐out approach was used.

### Patient selection

Prospectively collected data from consecutive patients with medial compartmental knee disorders who underwent closing‐wedge high tibial osteotomy (CWHTO) between February 2009 and September 2017 at a single institution were retrospectively evaluated. The study period ended in September 2017 because the present analysis was restricted to patients treated using the same historical CWHTO planning strategy. After this period, our institutional indications and planning approach changed, including greater consideration of the upper limit of postoperative MPTA and alternative osteotomy strategies. Therefore, more recent cases were not added in order to maintain cohort homogeneity. Patients with medial compartmental knee disorders were eligible for this study, including medial‐type osteoarthritis (OA) and osteonecrosis of the medial femoral condyle. Patients with radiographic OA changes in the lateral femorotibial compartment were not considered eligible for CWHTO. Medial compartment OA severity was assessed using the Kellgren–Lawrence (KL) classification [8]. Patellofemoral OA was assessed on preoperative radiographs as part of routine clinical evaluation; however, it was not graded using the KL classification in this study, and no patients were excluded solely because of patellofemoral OA. All patients included in this study underwent CWHTO; no opening‐wedge HTO or femoral corrective osteotomy was included. Radiographic OA severity was evaluated in all patients, including those with OA and osteonecrosis, using the KL classification [8].

In general, patients aged ≤70 years were eligible for HTO. However, four patients aged ≥70 years underwent CWHTO after shared decision‐making between the patient and surgeon, based on activity level, preference for joint‐preserving surgery and clinical suitability for osteotomy. No restriction was applied to body mass index (BMI). The choice of osteotomy technique was based on the planned correction angle and concomitant procedures. Opening‐wedge HTO was generally indicated for cases requiring ≤10° of correction. However, even when ≤10° of correction was planned, CWHTO was selected when concomitant osteochondral grafting was required. At the time of surgery, preoperative planning primarily aimed to achieve a postoperative mechanical axis (MA) of 65% through tibial correction alone. An upper limit for postoperative MPTA was not routinely applied, and femoral corrective osteotomy was not considered in the planning strategy.

### Surgical technique and postoperative rehabilitation

CWHTO was performed proximal to the tibial tuberosity, with the wedge size determined to achieve a postoperative MA of 65%. A 2‐cm segment of the fibula was resected at the midshaft. The osteotomy site was stabilised using the High Tibial Osteotomy System (Zimmer Biomet). All procedures were performed by the same surgical team (four surgeons). Postoperatively, the knee was immobilised with a brace for 1 week, and range‐of‐motion exercises were initiated thereafter. Partial weight bearing was permitted at 2 weeks postoperatively, and full weight bearing was allowed at 5 weeks, depending on pain and radiographic healing. Return to work and full activities were individualized according to occupation, symptoms, radiographic bone healing and functional recovery.

### Radiographic assessment

**Figure 1:**
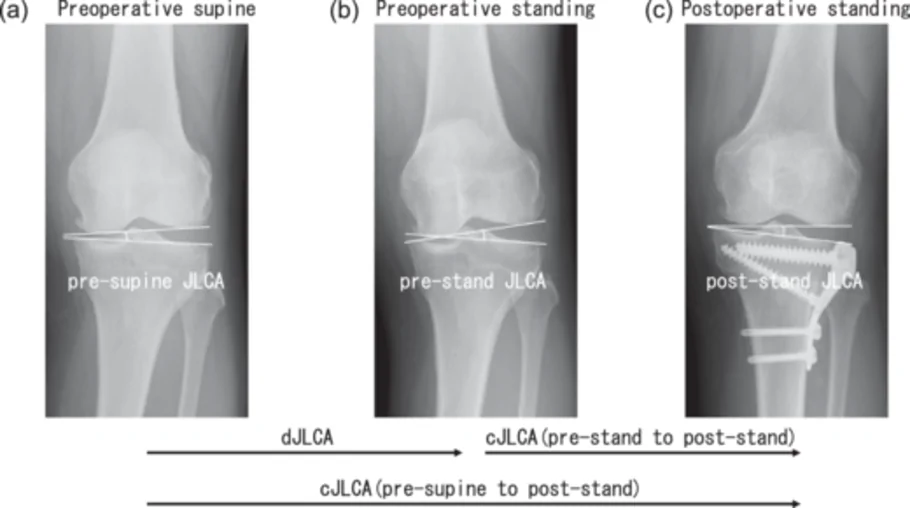
Representative illustration of preoperative standing JLCA, preoperative supine JLCA and postoperative standing JLCA. dJLCA was defined as preoperative standing JLCA minus preoperative supine JLCA. cJLCA (pre‐supine to post‐stand) was calculated as postoperative standing JLCA minus preoperative supine JLCA, and cJLCA (pre‐stand to post‐stand) was calculated as postoperative standing JLCA minus preoperative standing JLCA. (a) Preoperative supine. (b) Preoperative standing. (c) Postoperative standing. cJLCA, postoperative standing JLCA minus the indicated preoperative JLCA; dJLCA, preoperative standing JLCA minus preoperative supine JLCA; JLCA, joint line convergence angle.

Anteroposterior whole‐leg radiographs were obtained preoperatively in both the supine and standing positions. Postoperative standing whole‐leg radiographs were obtained during routine clinical follow‐up after patients were able to stand for radiographic assessment and bone union was considered clinically sufficient. Radiographs were considered non‐measurable and excluded if the femoral head, knee center or ankle center could not be identified, or if excessive limb rotation prevented reliable assessment. Standing radiographs were obtained with the patella facing forward as much as possible to minimize rotational inconsistency. In addition, preoperative anteroposterior knee radiographs were acquired under 150‐N varus and valgus stress with the knee flexed at approximately 15° using a Telos device (Metax). Varus and valgus stress radiographs were obtained to assess coronal‐plane joint laxity as a surrogate of soft‐tissue balance. JLCA measured under varus and valgus stress was used to reflect compartment opening under stress and was included as a preoperative factor that could influence postoperative cJLCA. The hip–knee–ankle (HKA) angle, lateral distal femoral angle (LDFA), MPTA and JLCA were measured using established methods [2, 18]. If the apex of the JLCA was medial, it was recorded as positive (varus), and if lateral, it was recorded as negative (valgus). The difference between preoperative standing and supine JLCA was defined as dJLCA. Postoperative change in JLCA was defined as cJLCA (pre‐supine to post‐stand) = post‐stand JLCA − pre‐supine JLCA and cJLCA (pre‐stand to post‐stand) = post‐stand JLCA − pre‐stand JLCA (Figure 1). All radiographic parameters were measured using a digital radiographic measurement tool (ShadeQuest/ViewR v1.29; FUJIFILM Medical Solutions Corporation).

### Statistical analysis

Associations between demographic and preoperative radiographic variables and the two outcomes were evaluated: cJLCA (pre‐supine to post‐stand) and cJLCA (pre‐stand to post‐stand). Spearman's rank correlation was used for univariate analyses. Multivariable linear regression models included age, BMI, varus and valgus stress JLCA and variables significant in the univariate analyses. Regression coefficients are reported with 95% confidence intervals (CIs). As a sensitivity analysis, the primary multivariable analyses were repeated after excluding patients whose postoperative standing whole‐leg radiographs were obtained before 6 months postoperatively to assess the potential influence of early postoperative standing radiographs.

An exploratory subgroup analysis was performed according to the postoperative standing mechanical axis percentage (%MA). Based on previously reported criteria by Lee et al. [5], knees were classified into three groups: undercorrection (%MA < 57%), acceptable correction (%MA 57%–67%) and overcorrection (%MA > 67%). JLCA‐related parameters were compared among the three groups using the Kruskal–Wallis test. When a significant difference was observed, post hoc pairwise comparisons were performed using the Mann–Whitney *U* test with Holm adjustment. In addition, Spearman's rank correlation coefficients were calculated to assess the associations between dJLCA and cJLCA within each subgroup. The preoperative standing and supine JLCA were compared with the midpoint of the varus and valgus stress JLCA values using the Wilcoxon signed‐rank test.

### Reliability assessment

Inter‐ and intra‐observer reliabilities were assessed only for HKA and JLCA measured on preoperative standing and supine whole‐leg radiographs. Inter‐observer reliability was quantified using intraclass correlation (ICC)(2,1) (two‐way random‐effects model, absolute agreement, single measurement) and intra‐observer reliability was quantified using ICC(3,1) (two‐way mixed‐effects model, absolute agreement, single measurement). Ninety‐five percent CIs were calculated for all ICCs.

## RESULTS

A total of 52 consecutive patients were screened. After excluding patients who underwent concomitant anterior cruciate ligament reconstruction (*n* = 1) and those with unavailable (*n* = 7) or non‐measurable radiographs (*n* = 4), 40 patients were included in the final analysis. Baseline characteristics are summarized in Table 1. The cohort comprised 29 women and 11 men with a mean age of 60.3 years and a mean BMI of 26.2 kg/m^2^. Postoperative whole‐leg radiographs were obtained at a mean of 9.9 months (range, 2–14 months). The underlying pathology included OA (*n* = 24) and osteonecrosis (*n* = 16). Medial compartment OA severity was graded using the KL grading (Grade II, *n* = 12; Grade III, *n* = 14; Grade IV, *n* = 14). Standardized patellofemoral compartment KL grading was not available in this retrospective dataset. Concomitant osteochondral grafting was performed in all osteonecrosis cases (*n* = 16), and one patient had a history of partial meniscectomy.

**Table 1:** Patient demographics.

Characteristic	Value
Pathology, *n*	
Osteoarthritis	24
Osteonecrosis	16
Sex, *n*	
Female	29
Male	11
Age, mean (range), years	60.3 (39–77)
BMI, mean (range), kg/m^2^	26.2 (20.1–36.3)
Postoperative radiographic follow‐up, mean (range), months	9.9 (2–14)
Medial compartment Kellgren–Lawrence grade, *n*	
II	12
III	14
IV	14
Concomitant osteochondral grafting, *n*	16
History of partial meniscectomy, *n*	1

Abbreviations: BMI, body mass index; kg, kilograms; *n*, number of patients.

Pre‐ and postoperative radiological parameters are summarised in Table 2. The mean planned correction angle was not significantly different from the actual change in MPTA (*p* = 0.094).

**Table 2:** Radiographic alignment measurements.

Parameter	Preoperative (supine), mean (range)	Preoperative (standing), mean (range)	Postoperative (standing), mean (range)	Change, mean (range)
LDFA (°)	—	89.0 (85.5–95.2)	—	—
MPTA (°)	—	84.3 (79.8–88.6)	95.8 (90.3–102.9)	11.4 (5.0–16.6)
HKA (°)	6.2 (1.2–12.1) varus	8.1 (1.6–12.7) varus	4.3 (−1.8 to 11.0) valgus	12.4 (6.5–20.1)^a^

Abbreviations: HKA, hip–knee–ankle angle; LDFA, lateral distal femoral angle; MPTA, medial proximal tibial angle.

^a^ Postoperative standing HKA minus preoperative standing HKA.

**Figure 2:**
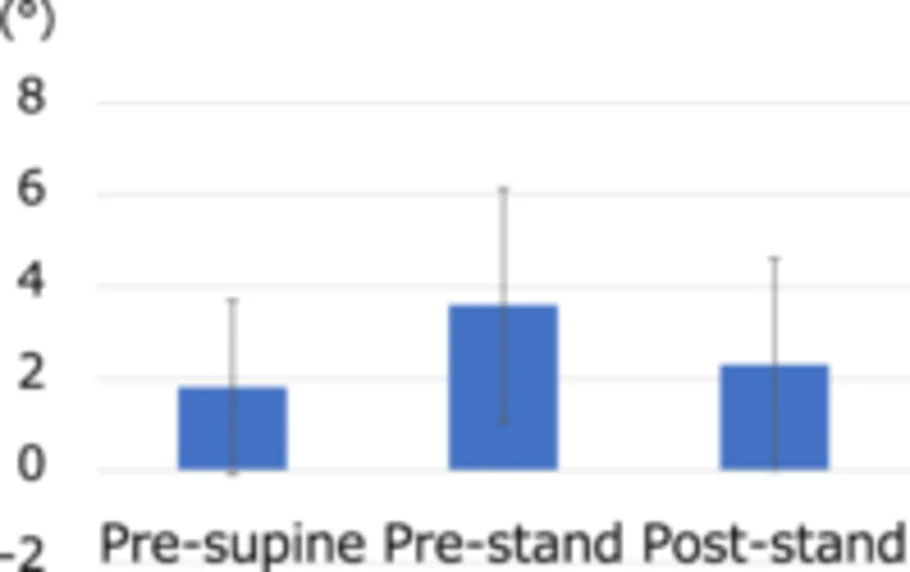
JLCA under various conditions (°). Error bars represent standard deviation. JLCA, joint line convergence angle.

JLCA‐related measurements are summarized in Table 3 and illustrated in Figure 2. The mean preoperative JLCA was 1.8° in the supine position and 3.6° in the standing position, with a mean dJLCA of 1.78°. The mean JLCA values under varus and valgus stress were 6.58° and −1.10°, respectively, and the mean stress‐view midpoint was 2.74°. Compared with the stress‐view midpoint, the preoperative supine JLCA was 0.92° more valgus (*p* = 0.022), whereas the preoperative standing JLCA was 0.86° more varus (*p* = 0.224) (Table 3).

**Table 3:** JLCA measurements.

Parameter	Mean (range)
Preoperative supine JLCA (°)	1.82 (−0.65 to 7.22)
Preoperative standing JLCA (°)	3.60 (0.81–10.39)
Preoperative varus stress JLCA (°)	6.58 (1.00–13.00)
Preoperative valgus stress JLCA (°)	−1.10 (−4.90 to 2.10)
Stress‐view midpoint (°)^a^	2.74 (−1.40 to 6.95)
Preoperative supine JLCA − stress‐view midpoint (°)	−0.92 (−4.12 to 3.02)
Preoperative stand JLCA − stress‐view midpoint (°)	0.86 (−3.06 to 4.79)
Postoperative standing JLCA (°)	2.31 (−1.74 to 8.64)
dJLCA (°)	1.78 (0.03–4.67)
cJLCA (pre–supine to post–stand, °)	0.49 (−2.18 to 4.77)
cJLCA (pre–stand to post–stand, °)	−1.29 (−3.50 to 1.76)

Abbreviations: cJLCA, postoperative standing JLCA minus the indicated preoperative JLCA; dJLCA, preoperative standing JLCA minus preoperative supine JLCA; JLCA, joint line convergence angle.

^a^ Stress‐view midpoint = (varus stress JLCA + valgus stress JLCA)/2.

Inter‐ and intra‐observer reliabilities for radiographic measurements were excellent. Inter‐observer ICC(2,1) values ranged from 0.964 to 0.971, with 95% CIs ranging from 0.927 to 0.988. Intra‐observer ICC(3,1) values ranged from 0.935 to 0.978, with 95% CIs ranging from 0.850 to 0.992.

The relationship between cJLCA and preoperative variables was examined. Univariate regression analysis showed a significant positive correlation between cJLCA (pre–supine to post–stand) and dJLCA (*ρ* = 0.52, *p* = 0.001), and significant negative correlation between cJLCA (pre–stand to post–stand) and both dJLCA (*ρ* = −0.43, *p* = 0.007) and preoperative standing JLCA (*ρ* = −0.41, *p* = 0.009) (Table 4).

**Table 4:** Spearman's correlation analysis of preoperative factors associated with cJLCA.

Variable	cJLCA (pre–supine to post–stand) *ρ* (*p* value)	cJLCA (pre–stand to post–stand) *ρ* (*p* value)
Age	0.14 (0.407)	0.21 (0.206)
BMI	0.14 (0.383)	−0.09 (0.599)
pre supine HKA	−0.02 (0.892)	−0.08 (0.609)
pre stand HKA	0.25 (0.133)	−0.10 (0.531)
pre LDFA	−0.09 (0.583)	0.05 (0.780)
pre MPTA	0.14 (0.410)	0.13 (0.423)
pre supine JLCA	0.06 (0.710)	−0.17 (0.315)
pre stand JLCA	0.28 (0.085)	−0.41 (0.009)*
dJLCA	0.52 (0.001)*	−0.43 (0.007)*
varus JLCA	0.25 (0.120)	−0.02 (0.892)
valgus JLCA	0.22 (0.184)	0.10 (0.555)

*Note*: *p* values were rounded to three decimal places.

Abbreviations: BMI, body mass index; cJLCA, change in joint line convergence angle; dJLCA, difference in the joint line convergence angle between the preoperative standing and supine positions; HKA, hip–knee–ankle angle; LDFA, lateral distal femoral angle; MPTA, medial proximal tibial angle; pre, preoperative supine radiograph; pre stand, preoperative standing radiograph; valgus, preoperative valgus radiograph; varus, preoperative varus stress radiograph; *ρ*, Spearman's rank correlation coefficient.

* Statistically significant (*p* < 0.05).

**Figure 3:**
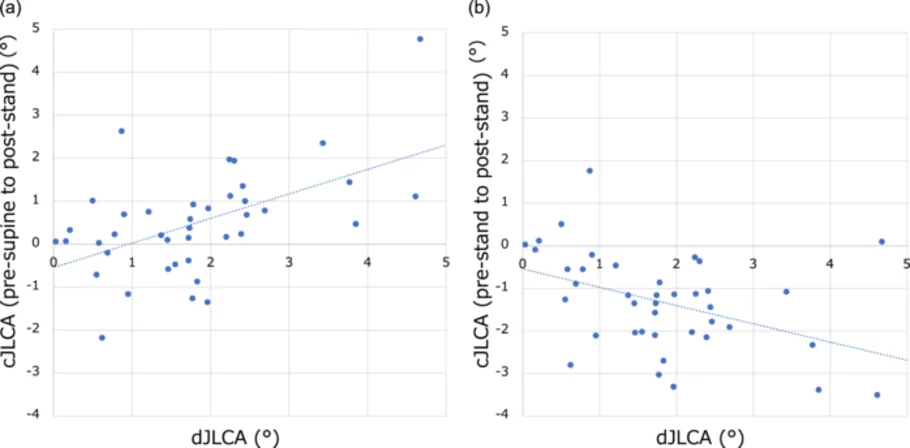
Relationship between dJLCA and cJLCA according to different planning radiographic references. Scatter plots show the correlation between dJLCA and cJLCA. Dotted lines represent linear regression fits. (a) A positive correlation was observed between dJLCA and cJLCA (postoperative standing JLCA minus preoperative supine JLCA), indicating that a greater dJLCA is associated with larger positive cJLCA. (b) A negative correlation was observed between dJLCA and cJLCA (postoperative standing JLCA minus preoperative standing JLCA), indicating that a greater dJLCA is associated with larger negative cJLCA. cJLCA, change in the joint line convergence angle; dJLCA, difference in the joint line convergence angle between the preoperative standing and supine positions; JLCA, joint line convergence angle.

In the multivariable regression analysis, dJLCA was positively associated with cJLCA (pre–supine to post–stand) (*B* = 0.51, 95% CI 0.16–0.86, *p* = 0.006) and negatively associated with cJLCA (pre–stand to post–stand) (*B* = −0.49, 95% CI −0.84 to −0.14, *p* = 0.008) (Table 5, Figure 3).

**Table 5:** Multiple regression analysis of preoperative factors associated with cJLCA.

Variable	cJLCA (pre–supine to post–stand), *B* (95% CI)	*p* value	cJLCA (pre–stand to post–stand), *B* (95% CI)	*p* value
Age	0.03 (−0.02 to 0.08)	0.246	0.03 (−0.02 to 0.08)	0.246
BMI	−0.03 (−0.14 to 0.08)	0.601	−0.03 (−0.14 to 0.08)	0.601
dJLCA	0.51 (0.16 to 0.86)	0.006*	−0.49 (−0.84 to −0.14)	0.008*
Varus stress JLCA	0.04 (−0.14 to 0.22)	0.651	0.04 (−0.14 to 0.22)	0.651
Valgus stress JLCA	0.08 (−0.15 to 0.31)	0.497	0.08 (−0.15 to 0.31)	0.491

Abbreviations: *B*, unstandardized regression coefficient; BMI, body mass index; CI, confidence interval; cJLCA, change in joint line convergence angle; dJLCA, preoperative standing JLCA minus preoperative supine JLCA; JLCA, joint line convergence angle.

* Statistically significant (*p* < 0.05).

In the exploratory subgroup analysis based on postoperative standing %MA, 7 knees (17.5%) were classified as undercorrected, 11 knees (27.5%) as acceptably corrected and 22 knees (55.0%) as overcorrected. Thus, only 27.5% of knees achieved acceptable correction in this cohort. No significant differences were observed in dJLCA or either cJLCA outcome among the three groups. In the overcorrection group, dJLCA was positively correlated with cJLCA (pre‐supine to post‐stand) (*ρ* = 0.656, *p* = 0.001). Because the subgroup sizes were small and unequal, these findings should be interpreted as exploratory (Supporting Information S1: Table S1).

Four patients had postoperative standing whole‐leg radiographs obtained before 6 months postoperatively and were excluded from the sensitivity analysis. In the remaining 36 patients, the associations between dJLCA and both cJLCA outcomes were directionally consistent with the primary analysis. dJLCA showed a positive association with cJLCA (pre‐supine to post‐stand), although this did not reach statistical significance (*B* = 0.33, 95% CI −0.04 to 0.69, *p* = 0.079). In contrast, dJLCA remained significantly negatively associated with cJLCA (pre‐stand to post‐stand) (*B* = −0.67, 95% CI −1.04 to −0.31, *p* = 0.001) (Supporting Information S1: Table S2).

## DISCUSSION

The principal finding was that dJLCA was independently associated with postoperative change in JLCA, and that this association depended on the radiographic condition used for preoperative planning. When supine whole‐leg radiographs were used as the reference, dJLCA was positively associated with cJLCA, suggesting a tendency toward undercorrection by approximately 50% of the dJLCA. In contrast, when standing whole‐leg radiographs were used as the reference, dJLCA was negatively associated with cJLCA, suggesting a tendency toward overcorrection by approximately 50% of the dJLCA. These findings suggest that dJLCA may be useful for anticipating soft‐tissue‐related alignment changes after CWHTO.

A mismatch between the planned and achieved postoperative alignment after HTO has been consistently reported [11, 13, 14, 19, 20, 21, 22, 23, 25]. Postoperative alignment reflects the combined effects of bony correction and changes in soft‐tissue balance [16]. Conventional preoperative planning focuses primarily on the intended change in MPTA and does not explicitly account for the soft‐tissue balance. Because JLCA can reflect coronal soft‐tissue balance [24], postoperative change in JLCA may contribute to unintended alignment and correction error [3, 11, 13, 14, 19, 20, 23, 25]. Therefore, identifying preoperative factors associated with cJLCA is clinically relevant for improving planning accuracy.

Only a few studies have examined determinants of postoperative change in JLCA after CWHTO, whereas most available evidence comes from open‐wedge high tibial osteotomy (OWHTO). Previous studies have identified preoperative standing JLCA, valgus JLCA, varus JLCA, joint‐space width differences, OA grade and dJLCA as factors associated with cJLCA or correction error [3, 11, 14, 19, 20, 21, 22, 23, 25]. In the present study, dJLCA emerged as the principal determinant of cJLCA in CWHTO, even after adjustment for valgus and varus stress JLCA. This finding is consistent with prior reports emphasizing the importance of dJLCA in predicting unintended alignment change [14, 21, 22]. To our knowledge, this is the first study to evaluate the determinants of cJLCA while directly comparing standing‐ and supine‐based planning in CWHTO.

The optimal radiographic condition for HTO planning remains debated. Weight‐bearing conditions are known to alter lower‐limb alignment and JLCA [7, 26], and several studies have recommended supine radiographs or individualized radiographic selection for preoperative planning [4, 12, 17, 21]. These recommendations are generally based on the concept that preoperative supine JLCA is closer to postoperative standing JLCA than preoperative standing JLCA. However, the present findings indicate that preoperative supine JLCA does not necessarily equal postoperative standing JLCA in all cases. Rather than suggesting that one radiographic condition is universally superior, the present results indicate that surgeons should recognize that postoperative change in JLCA differs according to the preoperative reference radiograph. In this context, dJLCA may serve as a practical indicator for anticipating cJLCA regardless of whether standing or supine whole‐leg radiographs are used. This study has several clinical implications. First, dJLCA can be calculated from routine supine and standing anteroposterior knee radiographs without special imaging such as stress radiography. Second, because supine whole‐leg radiographs may not be available in all institutions, the present findings may also be useful when planning is based only on standing whole‐leg radiographs. Third, approximately 50% of dJLCA may be considered when anticipating postoperative change in JLCA. However, this estimate should be interpreted cautiously because the sample size was small, and its clinical application requires validation in larger cohorts.

The exploratory subgroup analysis provided additional descriptive information regarding the direction of correction error. Acceptable correction was achieved in only 27.5% of knees in this cohort. This low proportion should be interpreted in the context of the historical planning strategy used in this cohort, which primarily targeted the planned %MA and did not explicitly incorporate postoperative soft‐tissue–related alignment changes, such as changes in JLCA. In the overcorrection group, dJLCA was significantly positively correlated with cJLCA when referenced to preoperative supine JLCA. Although the other subgroup correlations did not reach statistical significance, the directions of association in the acceptable correction and overcorrection groups were generally consistent with the overall analysis. These findings suggest that dJLCA may be useful for anticipating the direction of correction error, particularly excessive correction. However, because the subgroup sizes were small and unequal, these results should be interpreted as exploratory and hypothesis‐generating.

## LIMITATIONS

Some limitations should be acknowledged. First, the sample size was relatively small because the number of patients who underwent CWHTO at our institution during the study period was limited. Therefore, the number of variables included in the multivariable analysis had to be restricted to reduce the risk of overfitting. The present findings should be interpreted as exploratory and validated in larger multicenter studies. Second, the findings should be interpreted in the context of the historical planning strategy used in this cohort. Correction planning primarily aimed to achieve the target %MA through tibial correction alone, without routinely applying an upper limit for postoperative MPTA or considering femoral corrective osteotomy. Consequently, the mean postoperative MPTA was 95.7°, indicating substantial valgus correction of the proximal tibia. Although an MPTA of approximately 95° is commonly used as a threshold for excessive tibial valgus correction [1] and increased joint‐line obliquity [27], its clinical relevance remains controversial, and it should not be considered an absolute cutoff for poor outcomes [28]. Therefore, the present findings should not be interpreted as supporting routine excessive tibial correction and may not directly apply to current osteotomy planning strategies that incorporate an upper limit for MPTA or femoral corrective osteotomy. Third, this study included only patients who underwent CWHTO and comprised both OA and osteonecrosis cases; therefore, the generalizability of the findings to OWHTO or more homogeneous cohorts may be limited. Fourth, postoperative radiographs were obtained at variable time points, reflecting routine clinical follow‐up rather than a standardized assessment schedule. Although sensitivity analysis excluding patients with postoperative radiographs obtained before 6 months showed directionally consistent results, early postoperative standing alignment may still be influenced by pain, muscle weakness and incomplete functional recovery. Smoking status and standardized patellofemoral compartment KL grading were not available in our dataset. Finally, although inter‐ and intra‐observer reliabilities were excellent, small differences in JLCA‐related measurements on the order of 1°–2° may still fall within expected radiographic measurement variability and should therefore be interpreted cautiously.

## CONCLUSIONS

The preoperative standing‐supine difference in JLCA was independently associated with postoperative change in JLCA after CWHTO. A greater standing‐supine difference was associated with a larger positive postoperative change in JLCA when planning was referenced to supine whole‐leg radiographs, suggesting greater undercorrection. In contrast, when planning was referenced to standing whole‐leg radiographs, a greater standing‐supine difference was associated with a larger negative postoperative change in JLCA, suggesting greater overcorrection. These findings suggest that considering the standing‐supine difference in JLCA may help anticipate soft‐tissue‐related alignment changes after CWHTO. However, given the small sample size, measurement variability, historical planning strategy and variable timing of postoperative radiographs used in this cohort, the clinical application of these findings requires validation in larger studies.

## AUTHOR CONTRIBUTIONS

Mitsuhiko Kubo conceived and planned the study. Mitsuhiko Kubo and Kazuhiro Uenaka performed radiological measurements. Mitsuhiko Kubo, Kazuhiro Uenaka, Hitomi Fujikawa, Tsutomu Maeda, Kosuke Kumagai, Yuki Nosaka and Sadafumi Horikawa performed the surgery. Mitsuhiko Kubo took the lead in writing the manuscript. Shinji Imai supervised the study. All authors provided critical feedback and helped shape the research, analysis and manuscript.

## FUNDING INFORMATION

The authors have no funding to report.

## CONFLICT OF INTEREST STATEMENT

The authors declare no conflicts of interest.

## ETHICS STATEMENT

This retrospective study was approved by the Ethics Committee of Shiga University of Medical Science (No. R2025‐007). This study was conducted using an opt‐out consent process, as approved by the institutional review board. Information about the study was made publicly available, and participants were given the opportunity to decline participation.

## Supporting information

Supplementary Table S1. Exploratory subgroup analysis of JLCA‐related parameters according to postoperative standing mechanical axis percentage. Supplementary Table S2. Sensitivity analysis excluding patients with postoperative standing whole‐leg radiographs obtained before 6 months.

## Data Availability

The datasets generated and/or analysed during the current study are not publicly available, but are available from the corresponding author on reasonable request.
