# Supplementary material for: Preoperative standing–supine difference in joint line convergence angle predicts postoperative alignment change after closing‐wedge high tibial osteotomy
Source: J Exp Orthop. 2026 Sep 7;13(3):e70904. doi: 10.1002/jeo2.70904 (PMC13549370; doi:10.1002/jeo2.70904)
Supplement: Supplementary file 1 [file JEO2-13-e70904-s001.docx]

Supplementary Table S1. Exploratory subgroup analysis of JLCA-related parameters according to postoperative standing mechanical axis percentage

| **Group** | **Definition** | **n (%)** |
| --- | --- | --- |
| Undercorrection | %MA <57% | 7 (17.5%) |
| Acceptable correction | %MA 57–67% | 11 (27.5%) |
| Overcorrection | %MA >67% | 22 (55.0%) |

| **Parameter** | **Undercorrection (<57%), n=7** | **Acceptable correction (57–67%), n=11** | **Overcorrection (>67%), n=22** | **P value** |
| --- | --- | --- | --- | --- |
| Preoperative supine JLCA (°) | 2.56 (1.09 to 7.22) | 0.90 (−0.65 to 6.54) | 1.21 (0.09 to 4.00) | 0.063 |
| Preoperative standing JLCA (°) | 3.62 (1.59 to 9.18) | 1.90 (0.81 to 10.39) | 2.88 (0.84 to 5.83) | 0.360 |
| Postoperative standing JLCA (°) | 3.61 (2.10 to 7.27) | 1.30 (−1.23 to 8.64) | 1.34 (−1.74 to 4.21) | **0.016** |
| dJLCA (°) | 1.96 (0.50 to 2.46) | 1.55 (0.03 to 4.67) | 1.73 (0.16 to 4.61) | 0.992 |
| cJLCA (pre-supine to post-stand, °) | 1.00 (−1.35 to 2.63) | 0.69 (−0.58 to 4.77) | 0.19 (−2.18 to 1.94) | 0.298 |
| cJLCA (pre-stand to post-stand, °) | −0.55 (−3.31 to 1.76) | −0.86 (−3.38 to 0.12) | −1.46 (−3.50 to −0.09) | 0.091 |
| Varus stress JLCA (°) | 6.68 (2.89 to 12.70) | 7.10 (3.10 to 13.00) | 5.65 (1.00 to 9.50) | 0.241 |
| Valgus stress JLCA (°) | 0.00 (−3.00 to 1.80) | −1.50 (−4.30 to 2.10) | −0.99 (−4.90 to 1.50) | 0.247 |

| **Group** | **n** | **dJLCA vs cJLCA (pre-supine to post-stand), ρ (P value)** | **dJLCA vs cJLCA (pre-stand to post-stand), ρ (P value)** |
| --- | --- | --- | --- |
| Undercorrection | 7 | −0.107 (0.819) | −0.571 (0.180) |
| Acceptable correction | 11 | 0.600 (0.051) | −0.418 (0.201) |
| Overcorrection | 22 | **0.656 (0.001)** | −0.386 (0.076) |

Supplementary Table S2. Sensitivity analysis excluding patients with postoperative standing whole-leg radiographs obtained before 6 months

| **Outcome** | **B** | **95% CI** | **P value** |
| --- | --- | --- | --- |
| cJLCA (pre-supine to post-stand) | 0.33 | −0.04 to 0.69 | 0.079 |
| cJLCA (pre-stand to post-stand) | −0.67 | −1.04 to −0.31 | 0.001 |
